# Unsuspected foreign-body aspiration in adult patient with status asthmaticus: Case report

**DOI:** 10.1016/j.amsu.2020.05.026

**Published:** 2020-06-08

**Authors:** Liliana Fernandez-Trujillo, Valeria López-Castilla, Eliana I. Morales, Valeria Zúñiga-Restrepo, Diego F. Bautista

**Affiliations:** a Department of Internal Medicine, Pulmonology Service, Interventional Pulmonology, Fundación Valle del Lili, Cali, Colombia; b Faculty of Health Sciences, Department of Internal Medicine, Universidad Icesi, Cali, Colombia; c Department of Internal Medicine, Pulmonology Service, Fundación Valle del Lili, Cali, Colombia; d Clinical Research Center, Fundación Valle del Lili, Cali, Colombia; e Department of Critical Care Medicine, Fundación Valle del Lili, Cali, Colombia

**Keywords:** Asthma, Foreign bodies, Respiratory insufficiency, Bronchoscopy, Extracorporeal membrane oxygenation, Case report

## Abstract

**Introduction:**

Accidental foreign body aspiration can cause severe damage to the airway and threaten the patient's life. This situation requires multidisciplinary and systematic approach from the medical and surgical team, in order to achieve complete resolution maintaining airway permeability.

**Presentation of case:**

This is a 49 y/o man who presented with a severe asthma attack, in whom an unsuspected foreign body in the inferior airway was diagnosed, which was possibly the result of aspiration during the initial emergency care, causing worsening of the already critical condition.

**Discussion:**

We described the clinical course, radiologic and endoscopic findings, and outcome of the patient, highlighting the importance of considering the possibility of a foreign body in the airway, when there is no improvement in refractory status asthmaticus. This is particularly important in a university hospital. Moreover, the implementation of checklists when invasive procedures are performed can avoid loss of material, preventing iatrogenic aspiration events.

**Conclusion:**

Foreign body aspirations may remain undetected due to lack of suspicion, especially in adults, in whom they can cause chronic symptoms, or worsen chronic respiratory conditions turning them into more complex diseases. This cause must be considered in the differential diagnosis of refractory status asthmaticus.

## Highlights

• Accidental foreign body aspiration can cause severe damage to the airway.
• This situation can threaten the patient's life.
• Requires multidisciplinary and systematic approach from the medical and surgery team.
• The goal is to achieve complete resolution maintaining airway permeability.

## 1 Introduction

The era of bronchoscopy began with Gustav Killian in 1876 when he removed a pork bone from a farmer's airway, using an esophagoscope and cocaine as a topical anesthetic [1]. Since then, significant advances in both rigid and flexible bronchoscopy have been made. Foreign bodies in the lower respiratory tract are more frequent in children [2] but can also occur in adults and can result in a life-threatening event [3]. However, they rarely present with the triad of cough, dyspnea, and cyanosis [4]; instead, signs such as cough, hemoptysis, increased sputum production, wheezing and dyspnea or worsening of a chronic respiratory disease can be found [5]. The event may go unnoticed and be an incidental finding in bronchoscopy for chronic cough, hemoptysis or non-resolving pneumonia [6]. We present a case of a patient with refractory status asthmaticus worsened by a non-suspected foreign body of possible iatrogenic origin. This work has been reported in line with the SCARE criteria [7].

## 2 Presentation of Case

A 49-year-old man with a history of asthma that had been on short-acting inhaled bronchodilators and ipratropium bromide, with multiple hospitalizations in the last year due to asthmatic crises and poor outpatient control; presented with worsening of respiratory symptoms with cough, dyspnea, and wheezing of rapid onset. He was being treated for an asthmatic crisis on previous days and had been discharged with bronchodilator and anticholinergic inhalers 10 h prior to admission. He was assessed at a primary care center where they found respiratory distress, with retractions, sweating, no fever, no chest pain, no hemoptysis, with severely diminished breath sounds in both lungs and expiratory wheezing. Initially, a severe asthmatic crisis was diagnosed and orotracheal intubation was performed, with transfer to our hospital. He arrived in poor conditions, with difficulty for ventilation. Position of the endotracheal tube was verified and changed from a 7.0 to an 8.0 tube. Arterial blood gas analysis revealed hypoxemia, hypercapnia, and acidemia. Mechanical ventilation became difficult, with associated hyperinflation. The patient was initially administered intravenous (IV) magnesium sulfate, inhaled bronchodilators, IV epinephrine, and underwent muscle relaxation, with poor improvement of hypercapnia and acidosis. Veno-venous (VV) extracorporeal membrane oxygenation (ECMO) was early initiated, which guaranteed CO_2_ elimination and hemodynamic stability, as inflammation of the bronchi and secondary bronchospasm were continued to be treated with inhaled bronchodilators and IV steroids (hydrocortisone 100 mg q6H).

Other possible diagnoses were considered, due to non-improvement, such as carcinoid lesions, hypersensitivity pneumonitis, upper airway infections (UAI), and bacterial pneumonia, which can present as asthma mimickers in the acute setting [8]. Nonetheless, the patient had no image findings, nor risk factors for the first two etiologies. Such an acute onset of symptoms would unlike have been present in bacterial pneumonia or UAI, which are usually preceded by respiratory symptoms for about two weeks before worsening, especially in previously healthy patients. Given the patient's history of uncontrolled asthma, a status asthmaticus was considered our first and most likely workup diagnosis.

**Fig. 1:**
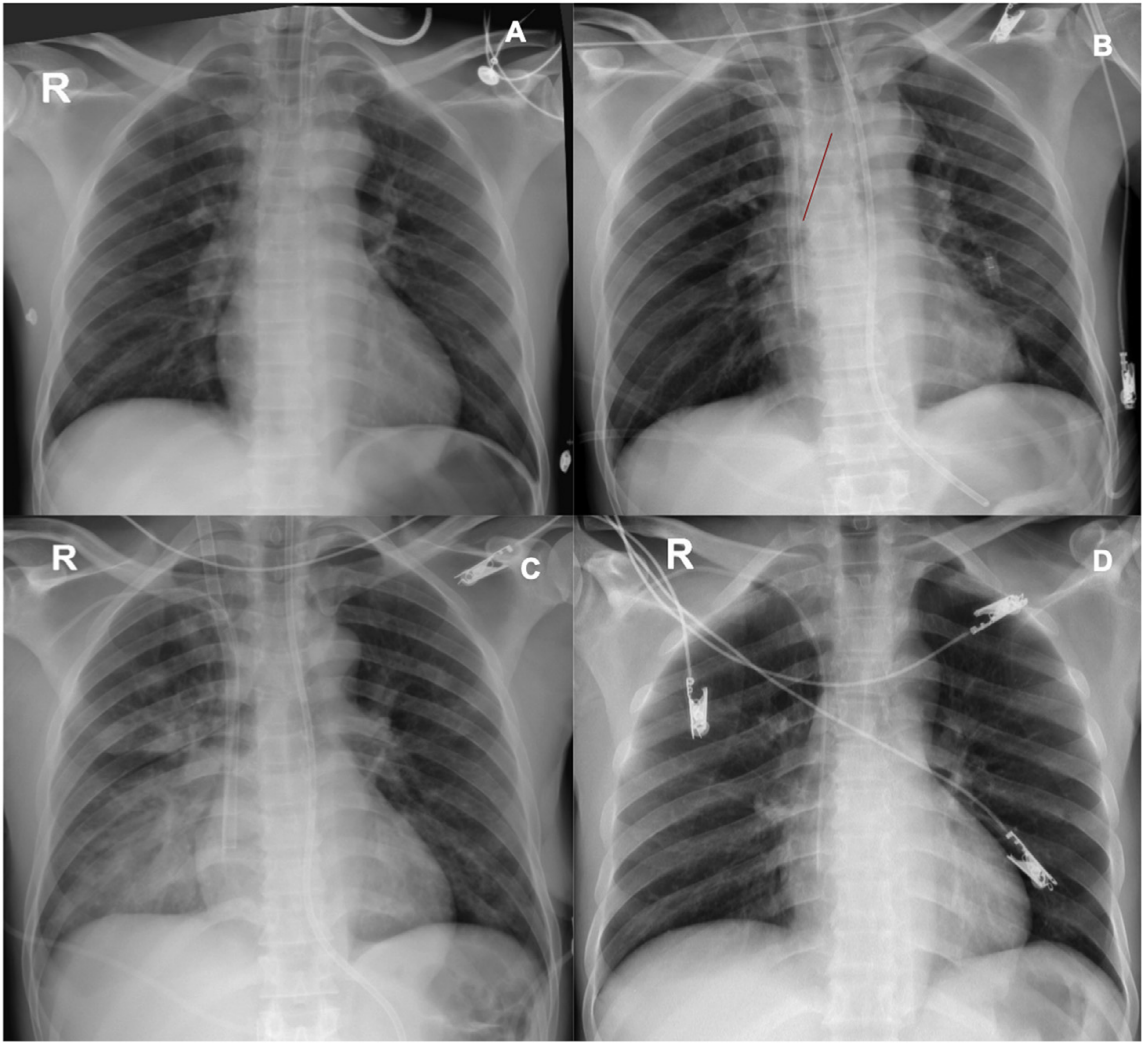
**A.** Anteroposterior chest X-ray taken at arrival where the orotracheal tube is in position, no pulmonary infiltrates, nor pleural effusion. **B.** Chest x-ray showing the jugular vein cannula for VV ECMO. The red line shows what was retrospectively identified as a possible foreign body in the airway. **C.** Chest x-ray with right lower lobe pulmonary alveolar infiltrates **D.** Chest x-ray at discharge without infiltrates or other alterations.

**Fig. 2:**
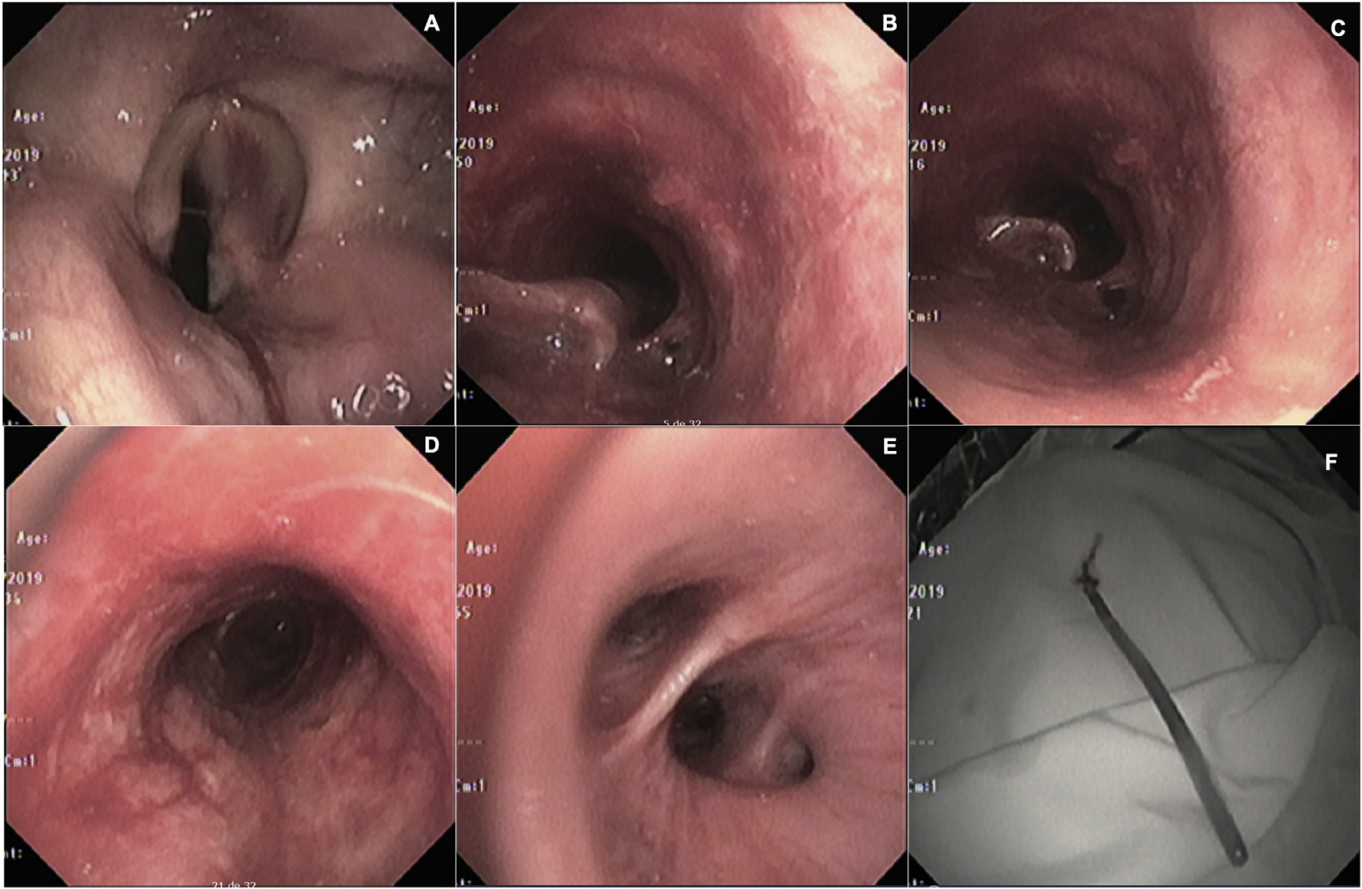
**A.** Vocal cords, right cord shows minor laceration. **B.** Upper trachea with edema and erythema of the mucosa and presence of the upper part of the foreign body, elongated structure with an irregular upper edge, mobile, lying on the posterior wall. **C.** Foreign body that moves down through the trachea during the respiratory cycle. **D.** Trachea with superficial lacerations on the posterior membrane, after removing the foreign body. **E.** Middle lobe and right lower lobe with mucosa, size, and normal branches. **F.** Extracted foreign body, hard plastic structure with a blunt and closed bottom tip of 12 cm long.

During hospitalization in the intensive care unit (ICU), progressive infiltrates in the right pulmonary base were detected, which were interpreted as a lower respiratory tract infection (Fig. 1). Antibiotics were started, and despite extubation was achieved, VV-ECMO was continued with lower oxygen requirements. Due to suspicion of pneumonia, bronchoscopy with bronchoalveolar lavage (BAL) was performed in the ICU, showing symmetric vocal cords with normal mobility, trachea with inflamed mucosa with edema and erythema, in the lumen of the trachea there was a non-suspected long foreign body that resembled a hard consistency feeding tube, aspiration probe or straw that moved during the respiratory cycle and entered the right main stem bronchus. It was extracted using claw clamps without complications. BAL was performed in the right lower lobe. The foreign body was an elongated and hard plastic structure of approximately 12 cm long similar to an aspiration probe. In Fig. 2, the vocal cords are identified (A), the trachea is seen with edema with the upper end of the foreign body approaching and moving away with the respiratory cycle (B, C). Despite mild edema, the trachea seems of normal appearance after the extraction of the foreign body (D. The middle and right lower lobe appear normal (E). Lastly, the unsuspected foreign body is shown: a semi-hard plastic element that was believed to be a part of respiratory therapy supplies, which was accidently introduced in the patient's airway during initial assessment.

After foreign body extraction, the patient had a better clinical evolution with lower oxygen requirement, for which ECMO was removed, and antibiotics were completed, as well as oral steroids with an improvement of symptoms and wheezing.

Asthma medication was adjusted, with prescription of a long-acting beta-agonist and inhaled steroid. During re-interrogation, no events that made the presence of a foreign body probable before arrival to the emergency department were found. The patient was discharged after a hospital stay of six days in the ICU, and four days in the intermediate care unit.

## 3 Discussion

Foreign bodies in the lower respiratory tract are more frequent in children, representing up to 85% of the cases [2]. However, in adults, they also occur and can result in life-threatening events [3]. Most commonly the aspiration of organic material such as meat, nuts or fish bones is found. However, materials such as glass, dental devices and metal objects are also described. The tissue reaction in the airway depends on the material [9]; inorganic materials cause little inflammation but can cause direct trauma to the airway, while organic compounds cause significant inflammation with granulation tissue resulting in stenosis [3].

We described a patient with multiple hospitalizations probably due to under-treatment (no inhaled steroid on treatment regimen) and recurrent asthmatic crises, one of them involving a status asthmaticus refractory to conventional management. The foreign body found during BAL performance was an inorganic material, similar to an aspiration probe, which probably resulted from respiratory therapy or intubation performed at the primary care center. In adults, iatrogenic etiology can account for up to 80% of cases of inorganic foreign bodies in the lower respiratory tract [10].

The preferred method in adults is both flexible and rigid bronchoscopy, which is relatively safe and easily carried out by an expert [6]. During the procedure, the airway is visualized, and the object is removed with claw clamps, baskets, magnetized clamps, as appropriate, usually without additional risks [10]. If the material is attached or granulation tissue is present, previous steroid treatment can be useful. If not, a surgical procedure with resection should be performed, in up to 10% of the cases, to avoid symptoms and long-term complications such as bleeding, intractable cough, and infections [11,12].

In this case, the foreign body origin was not for sure identified. However, given the clinical course and past medical history that made aspiration prior to admission highly unlikely, it was most probably introduced to the airway during respiratory therapy or intubation in the primary care center, since this material is not part of our hospital's equipment.

## 4 Conclusions

Unsuspected foreign body aspiration is an event that can occur in adults with variable onset presentation and should be considered as a differential diagnosis in the evaluation of complex and refractory cases. In adults, clinical presentation is not as clear as in children, for they can also be asymptomatic. Here, a severe status asthmaticus with the need for early ECMO was presented, and there was no evidence of the time of aspiration of the foreign body, but in the assessment of pulmonary infiltrates in a critical patient, bronchoscopy was key for identification and removal of the foreign body without complications.

This case points out the importance of considering the possibility of a foreign body in the airway as an alternative when there is no improvement in refractory status asthmaticus despite adequate therapeutic agents. Also, the implementation of checklists when procedures such as intubation or respiratory therapy are performed in the acutely ill patient can avoid loss of material, preventing iatrogenic aspiration events.

## Ethics approval and consent to participate

This report was prepared in accordance with the ethical standards of the institutional ethics committee and with the 1964 Helsinki Declaration. We have approval letter of Ethics Committee in biomedical research IRB/EC No. 390 - 2019 of the Fundación Valle del Lili to publish this manuscript.

## Consent for publication

Written informed consent was obtained from the patient for publication of this case report and any accompanying images. A copy of the written consent is available for review by the Editor-in-Chief of this journal.

## Availability of data and materials

All data and material are available for sharing if needed.

## Funding

No funding sources were used.

## Authors’ contributions

All authors contributed to data analysis, drafting and revising the article, gave final approval of the version to be published, and agree to be accountable for all aspects of the work.

## Provenance and peer review

Not commissioned, externally peer reviewed.

## Declaration of competing interest

The authors declare that they have no competing interests. This manuscript has not been published and is not under consideration for publication elsewhere. Additionally, all of the authors have approved the contents of this paper and have agreed to the journal's submission policies.

## Abbreviations

VV: Veno-venous
IV: Intravenous
ECMO: extracorporeal membrane oxygenation
CO2: Carbon dioxide
ICU: Intensive care unit
UAI: Upper Airway Infections
BAL: Bronchoalveolar lavage
